# 1-De­oxy-d-galactitol (l-fucitol)

**DOI:** 10.1107/S1600536808020345

**Published:** 2008-07-09

**Authors:** Sarah F. Jenkinson, K. Victoria Booth, Akihide Yoshihara, Kenji Morimoto, George W. J. Fleet, Ken Izumori, David J. Watkin

**Affiliations:** a Department of Organic Chemistry, Chemical Research Laboratory, University of Oxford, Mansfield Road, Oxford OX1 3TA, England; b Rare Sugar Research Centre, Kagawa University, 2393 Miki-cho, Kita-gun, Kagawa 761-0795, Japan; c Department of Chemical Crystallography, Chemical Research Laboratory, University of Oxford, Mansfield Road, Oxford OX1 3TA, England

## Abstract

1-De­oxy-d-galactitol, C_6_H_14_O_5_, exists in the crystalline form as hydrogen-bonded layers of mol­ecules running parallel to the *ac* plane, with each mol­ecule acting as a donor and acceptor of five hydrogen bonds.

## Related literature

For related literature, see: Yoshihara *et al.* (2008 ▶); Jones *et al.* (2007 ▶); Görbitz (1999 ▶); Izumori (2002 ▶, 2006 ▶); Prince (1982 ▶); Watkin (1994 ▶).
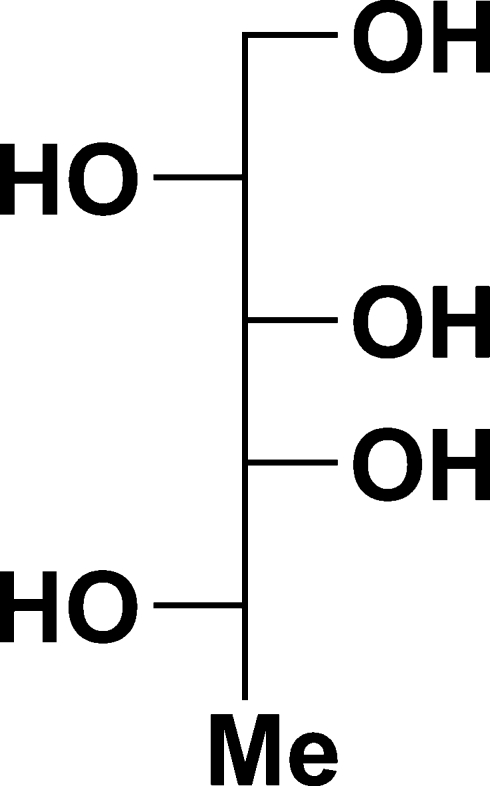


## Experimental

### 

#### Crystal data

C_6_H_14_O_5_
*M* *_r_* = 166.17
Monoclinic, 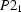

*a* = 4.8486 (3) Å
*b* = 4.8827 (3) Å
*c* = 16.8354 (13) Å
β = 92.856 (2)°
*V* = 398.07 (5) Å^3^
*Z* = 2
Mo *K*α radiation
μ = 0.12 mm^−1^
*T* = 150 K
0.15 × 0.15 × 0.05 mm

#### Data collection

Nonius KappaCCD diffractometer
Absorption correction: multi-scan (*DENZO*/*SCALEPACK*; Otwinowski & Minor, 1997 ▶) *T*_min_ = 0.81, *T*_max_ = 0.99
2786 measured reflections
998 independent reflections
804 reflections with *I* > 2σ(*I*)
*R*_int_ = 0.038

#### Refinement

*R*[*F* ^2^ > 2σ(*F* ^2^)] = 0.040
*wR*(*F* ^2^) = 0.111
*S* = 0.88
998 reflections
100 parameters
1 restraint
H-atom parameters constrained
Δρ_max_ = 0.34 e Å^−3^
Δρ_min_ = −0.31 e Å^−3^

### 

Data collection: *COLLECT* (Nonius, 2001 ▶); cell refinement: *DENZO*/*SCALEPACK* (Otwinowski & Minor, 1997 ▶); data reduction: *DENZO*/*SCALEPACK*; program(s) used to solve structure: *SIR92* (Altomare *et al.*, 1994 ▶); program(s) used to refine structure: *CRYSTALS* (Betteridge *et al.*, 2003 ▶); molecular graphics: *CAMERON* (Watkin *et al.*, 1996 ▶); software used to prepare material for publication: *CRYSTALS*.

## Supplementary Material

Crystal structure: contains datablocks global, I. DOI: 10.1107/S1600536808020345/lh2653sup1.cif

Structure factors: contains datablocks I. DOI: 10.1107/S1600536808020345/lh2653Isup2.hkl

Additional supplementary materials:  crystallographic information; 3D view; checkCIF report

## Figures and Tables

**Table 1:** Hydrogen-bond geometry (Å, °)

*D*—H⋯*A*	*D*—H	H⋯*A*	*D*⋯*A*	*D*—H⋯*A*
O4—H1⋯O6^i^	0.83	1.91	2.691 (4)	155
O9—H3⋯O4^ii^	0.83	1.97	2.753 (4)	156
O6—H4⋯O1^iii^	0.81	2.10	2.758 (4)	138
O1—H9⋯O9^iv^	0.85	1.85	2.684 (4)	166
O11—H10⋯O11^v^	0.84	2.01	2.828 (4)	163

Symmetry codes: (i) 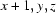; (ii) 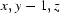; (iii) 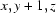; (iv) 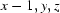; (v) 
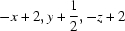
.

**Fig. 1.**
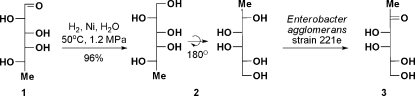
Synthetic scheme.

**Fig. 2.**
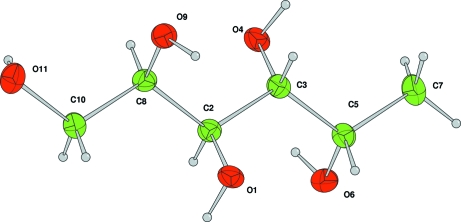
The title compound with displacement ellipsoids drawn at the 50% probability level. H atoms are shown as spheres of arbitary radius.

**Fig. 3.**
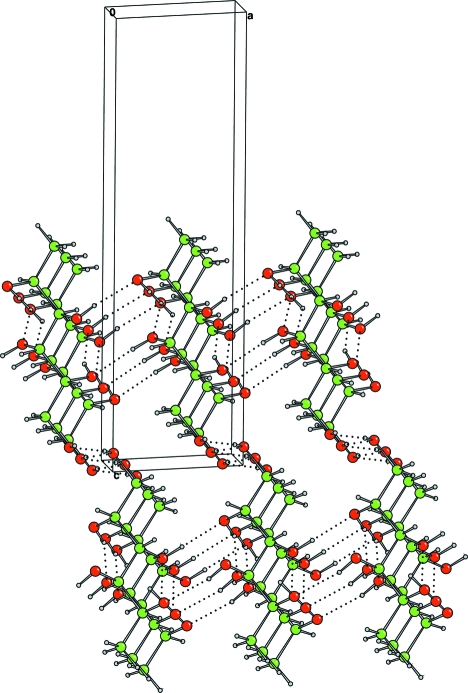
The packing diagram for the title compound projected along the b-axis. Hydrogen bonds are shown as dotted lines.

## supplementary crystallographic information

### Comment

The methodology developed by Izumori (2002, 2006) for the interconversion of tetroses, pentoses and hexoses by enzymatic oxidation, inversion at C3 with a single epimerase, and reduction to the aldose has been seen to be generally applicable for the 1-deoxy ketohexoses (Yoshihara *et al.*, 2008). This methodology could allow access to rare monosaccharides in water in large amounts. An example of this is the subsequent formation of 1-deoxy-D-galactitol 2 by hydrogenation of L-fucose 1 (Fig. 1) which subsequently could be oxidized enzymatically to 1-deoxy-D-tagatose (Jones *et al.*, 2007) 3.

If the terminal hydroxyl group and H atoms are ignored there is a pseudo centre of symmetry between C2 and C3 (Fig. 2). The crystal structure exists of hydrogen-bonded layers of molecules running parallel to the *c*-axis (Fig. 3). Each molecule acts as a donor and acceptor of 5 hydrogen bonds, all intra-molecular hydrogen bonds have been omitted.

### Experimental

The title compound was recrystallized from methanol: m.p. 420-422K; [α]_D_^21^ +1.6 (*c*, 1.13 in H_2_O) [Lit. (Yoshihara *et al.*, 2008) for enantiomer [α]_D_^20^ -1.9 (*c*, 1.0 in H_2_O)].

### Refinement

In the absence of significant anomalous scattering, Friedel pairs were merged and the absolute configuration was assigned fron the starting material.

The relatively large ratio of minimum to maximum corrections applied in the multiscan process (1:1.22 reflect changes in the illuminated volume of the crystal. Changes in illuminated volume were kept to a minimum, and were taken into account (Görbitz, 1999) by the multi-scan inter-frame scaling (*DENZO*/*SCALEPACK*, Otwinowski & Minor, 1997).

The H atoms were all located in a difference map, but those attached to carbon atoms were repositioned geometrically. The H atoms were initially refined with soft restraints on the bond lengths and angles to regularize their geometry (C—H in the range 0.93–0.98, O—H = 0.82 Å) and *U*_iso_(H) (in the range 1.2–1.5 times *U*_eq_ of the parent atom), after which the positions were refined with riding constraints.

### Crystal data

**Table:**

C_6_H_14_O_5_	*F*_000_ = 180
*M**_r_* = 166.17	*D*_x_ = 1.386 Mg m^−^^3^
Monoclinic, *P*2_1_	Mo *K*α radiation λ = 0.71073 Å
*a* = 4.8486 (3) Å	Cell parameters from 844 reflections
*b* = 4.8827 (3) Å	θ = 5–27º
*c* = 16.8354 (13) Å	µ = 0.12 mm^−^^1^
β = 92.856 (2)º	*T* = 150 K
*V* = 398.07 (5) Å^3^	Block, colourless
*Z* = 2	0.15 × 0.15 × 0.05 mm

### Data collection

**Table:**

Nonius KappaCCD diffractometer	804 reflections with *I* > 2σ(*I*)
Monochromator: graphite	*R*_int_ = 0.038
*T* = 150 K	θ_max_ = 27.4º
ω scans	θ_min_ = 5.4º
Absorption correction: multi-scan(DENZO/SCALEPACK; Otwinowski & Minor, 1997)	*h* = −6→6
*T*_min_ = 0.81, *T*_max_ = 0.99	*k* = −5→6
2786 measured reflections	*l* = −21→21
998 independent reflections	

### Refinement

**Table:**

Refinement on *F*^2^	Primary atom site location: structure-invariant direct methods
Least-squares matrix: full	Hydrogen site location: inferred from neighbouring sites
*R*[*F*^2^ > 2σ(*F*^2^)] = 0.040	H-atom parameters constrained
*wR*(*F*^2^) = 0.111	Method, part 1, Chebychev polynomial, (Watkin, 1994; *P*rince, 1982) [*w*eight] = 1.0/[A_0_*T_0_(x) + A_1_*T_1_(x) ··· + A_n-1_]*T_n-1_(x)] where A_i_ are the Chebychev coefficients listed belo*w* and x = *F* /*F*max Method = Robust Weighting (*P*rince, 1982) W = [*w*eight] * [1-(delta*F*/6*sigma*F*)^2^]^2^ A_i_ are: 17.0 25.0 12.0 3.16
*S* = 0.88	(Δ/σ)_max_ = 0.0002
998 reflections	Δρ_max_ = 0.34 e Å^−^^3^
100 parameters	Δρ_min_ = −0.31 e Å^−^^3^
1 restraint	Extinction correction: none

### Fractional atomic coordinates and isotropic or equivalent isotropic displacement parameters (Å^2^)

**Table:**

	*x*	*y*	*z*	*U*_iso_*/*U*_eq_	
O1	0.4779 (4)	0.0226 (5)	0.76245 (11)	0.0217	
C2	0.6328 (6)	0.2631 (7)	0.78168 (17)	0.0186	
C3	0.7866 (6)	0.3389 (7)	0.70769 (17)	0.0189	
O4	0.9430 (4)	0.5805 (5)	0.72728 (12)	0.0227	
C5	0.5946 (6)	0.3936 (7)	0.63490 (17)	0.0207	
O6	0.4117 (4)	0.6179 (5)	0.64879 (12)	0.0238	
C7	0.7550 (7)	0.4471 (9)	0.56067 (18)	0.0330	
C8	0.8283 (6)	0.2108 (7)	0.85426 (17)	0.0190	
O9	1.0094 (4)	−0.0141 (5)	0.84026 (12)	0.0222	
C10	0.6698 (6)	0.1572 (7)	0.92859 (17)	0.0236	
O11	0.8526 (4)	0.1176 (5)	0.99759 (12)	0.0260	
H21	0.5071	0.4100	0.7945	0.0249*	
H31	0.9082	0.1875	0.6971	0.0263*	
H51	0.4763	0.2307	0.6253	0.0282*	
H71	0.6272	0.4510	0.5138	0.0515*	
H72	0.8900	0.3047	0.5550	0.0518*	
H73	0.8493	0.6223	0.5674	0.0506*	
H81	0.9485	0.3709	0.8670	0.0243*	
H101	0.5642	−0.0123	0.9193	0.0325*	
H102	0.5415	0.3107	0.9363	0.0333*	
H1	1.0737	0.5438	0.6989	0.0372*	
H3	0.9415	−0.1296	0.8087	0.0364*	
H4	0.5121	0.7060	0.6789	0.0402*	
H9	0.3277	0.0397	0.7859	0.0353*	
H10	0.9076	0.2813	0.9992	0.0410*	

### Atomic displacement parameters (Å^2^)

**Table:**

	*U*^11^	*U*^22^	*U*^33^	*U*^12^	*U*^13^	*U*^23^
O1	0.0179 (9)	0.0228 (13)	0.0249 (10)	−0.0051 (9)	0.0048 (8)	−0.0061 (10)
C2	0.0180 (13)	0.0189 (15)	0.0189 (13)	−0.0011 (11)	0.0010 (10)	0.0011 (12)
C3	0.0196 (13)	0.0173 (15)	0.0202 (13)	−0.0016 (12)	0.0031 (11)	−0.0029 (12)
O4	0.0212 (10)	0.0235 (13)	0.0237 (9)	−0.0059 (10)	0.0057 (8)	−0.0040 (10)
C5	0.0210 (14)	0.0218 (17)	0.0196 (13)	0.0007 (13)	0.0029 (11)	−0.0017 (12)
O6	0.0188 (9)	0.0271 (13)	0.0254 (10)	0.0014 (10)	0.0003 (8)	−0.0008 (11)
C7	0.0320 (17)	0.048 (2)	0.0192 (14)	0.0027 (17)	0.0047 (12)	0.0033 (16)
C8	0.0166 (13)	0.0198 (15)	0.0204 (13)	0.0021 (12)	0.0006 (10)	−0.0004 (12)
O9	0.0206 (10)	0.0227 (12)	0.0233 (10)	0.0015 (10)	0.0011 (8)	−0.0047 (10)
C10	0.0223 (14)	0.031 (2)	0.0179 (13)	0.0020 (13)	0.0023 (11)	−0.0001 (13)
O11	0.0323 (11)	0.0248 (11)	0.0206 (9)	−0.0028 (11)	−0.0024 (8)	0.0022 (10)

### Geometric parameters (Å, °)

**Table:**

O1—C2	1.423 (4)	O6—H4	0.809
O1—H9	0.849	C7—H71	0.979
C2—C3	1.529 (4)	C7—H72	0.963
C2—C8	1.530 (4)	C7—H73	0.974
C2—H21	0.972	C8—O9	1.433 (4)
C3—O4	1.432 (4)	C8—C10	1.523 (4)
C3—C5	1.525 (4)	C8—H81	0.992
C3—H31	0.968	O9—H3	0.832
O4—H1	0.832	C10—O11	1.439 (4)
C5—O6	1.436 (4)	C10—H101	0.982
C5—C7	1.527 (4)	C10—H102	0.987
C5—H51	0.989	O11—H10	0.843
			
C2—O1—H9	105.6	C5—C7—H71	109.6
O1—C2—C3	106.7 (2)	C5—C7—H72	109.6
O1—C2—C8	110.0 (3)	H71—C7—H72	109.9
C3—C2—C8	112.6 (2)	C5—C7—H73	108.1
O1—C2—H21	109.2	H71—C7—H73	110.5
C3—C2—H21	109.8	H72—C7—H73	109.1
C8—C2—H21	108.5	C2—C8—O9	110.9 (2)
C2—C3—O4	106.6 (2)	C2—C8—C10	111.5 (2)
C2—C3—C5	113.2 (2)	O9—C8—C10	110.0 (3)
O4—C3—C5	109.6 (3)	C2—C8—H81	112.0
C2—C3—H31	106.9	O9—C8—H81	106.3
O4—C3—H31	110.6	C10—C8—H81	105.8
C5—C3—H31	109.8	C8—O9—H3	113.5
C3—O4—H1	95.8	C8—C10—O11	111.8 (2)
C3—C5—O6	111.1 (2)	C8—C10—H101	107.1
C3—C5—C7	111.9 (2)	O11—C10—H101	108.1
O6—C5—C7	110.3 (3)	C8—C10—H102	108.9
C3—C5—H51	108.5	O11—C10—H102	111.3
O6—C5—H51	106.4	H101—C10—H102	109.5
C7—C5—H51	108.6	C10—O11—H10	94.6
C5—O6—H4	98.7		

### Hydrogen-bond geometry (Å, °)

**Table:**

*D*—H···*A*	*D*—H	H···*A*	*D*···*A*	*D*—H···*A*
O4—H1···O6^i^	0.83	1.91	2.691 (4)	155
O9—H3···O4^ii^	0.83	1.97	2.753 (4)	156
O6—H4···O1^iii^	0.81	2.10	2.758 (4)	138
O6—H4···O4	0.81	2.29	2.842 (4)	126
O1—H9···O9^iv^	0.85	1.85	2.684 (4)	166
O11—H10···O11^v^	0.84	2.01	2.828 (4)	163

Symmetry codes: (i) *x*+1, *y*, *z*; (ii) *x*, *y*−1, *z*; (iii) *x*, *y*+1, *z*; (iv) *x*−1, *y*, *z*; (v) −*x*+2, *y*+1/2, −*z*+2.

## References

[bb1] Altomare, A., Cascarano, G., Giacovazzo, C., Guagliardi, A., Burla, M. C., Polidori, G. & Camalli, M. (1994). *J. Appl. Cryst.* **27**, 435.

[bb2] Betteridge, P. W., Carruthers, J. R., Cooper, R. I., Prout, K. & Watkin, D. J. (2003). *J. Appl. Cryst.* **36**, 1487.

[bb3] Görbitz, C. H. (1999). *Acta Cryst.* B**55**, 1090–1098. 10.1107/s0108768199008721 10927450

[bb4] Izumori, K. J. (2002). *Naturwissenschaften*, **89**, 120–124. 10.1007/s00114-002-0297-z 12046631

[bb5] Izumori, K. J. (2006). *Biotechnology*, **124**, 717–722. 10.1016/j.jbiotec.2006.04.016 16716430

[bb6] Jones, N. A., Jenkinson, S. F., Soengas, R., Izumori, K., Fleet, G. W. J. & Watkin, D. J. (2007). *Acta Cryst.* C**63**, o7–o10. 10.1107/S0108270106048591 17206062

[bb7] Nonius (2001). *COLLECT* Nonius BV, Delft, The Netherlands.

[bb8] Otwinowski, Z. & Minor, W. (1997). *Methods in Enzymology*, Vol. 276, *Macromolecular Crystallography*, Part A, edited by C. W. Carter Jr & R. M. Sweet, pp. 307–326. New York: Academic Press.

[bb9] Prince, E. (1982). *Mathematical Techniques in Crystallography and Materials Science* New York: Springer-Verlag.

[bb10] Watkin, D. (1994). *Acta Cryst.* A**50**, 411–437.

[bb11] Watkin, D. J., Prout, C. K. & Pearce, L. J. (1996). *CAMERON* Chemical Crystallography Laboratory, Oxford, England.

[bb12] Yoshihara, A., Haraguchi, S., Gullapalli, P., Rao, D., Morimoto, K., Takata, G., Jones, N., Jenkinson, S. F., Wormald, M. R., Dwek, R. A., Fleet, G. W. J. & Izumori, K. (2008). *Tetrahedron Asymmetry*, **19**, 739–745.

